# Developing a Time Series Predictive Model for Dengue in Zhongshan, China Based on Weather and Guangzhou Dengue Surveillance Data

**DOI:** 10.1371/journal.pntd.0004473

**Published:** 2016-02-19

**Authors:** Yingtao Zhang, Tao Wang, Kangkang Liu, Yao Xia, Yi Lu, Qinlong Jing, Zhicong Yang, Wenbiao Hu, Jiahai Lu

**Affiliations:** 1 Department of Medical Statistics and Epidemiology, School of Public Health, Sun Yat-sen University, Guangzhou, Guangdong Province, P. R. China; 2 Zhongshan Center for Disease Control and Prevention, Zhongshan, Guangdong Province, P. R. China; 3 Zhongshan Institute of School of Public Health, Sun Yat-sen University, Zhongshan, Guangdong Province, P. R. China; 4 Department of Environmental Health, School of Public Health, University at Albany, State University of New York, Albany, New York, United States of America; 5 Guangzhou Center for Disease Control and Prevention, Guangzhou, Guangdong Province, P. R. China; 6 School of Public Health and Social Work, Queensland University of Technology, Brisbane, Australia; 7 Institute of Health and Biomedical Innovation, Queensland University of Technology, Brisbane, Australia; 8 Key Laboratory for Tropical Diseases Control of Ministry of Education, Sun Yat-sen University, Guangzhou, Guangdong Province, P. R. China; 9 One Health Center of Excellence for Research and Training, School of Public Health, Sun Yat-sen University, Guangzhou, Guangdong Province, P. R. China; 10 Institute of Emergency Technology for Serious Infectious Diseases Control and Prevention, Guangdong Provincial Department of Science and Technology; Emergency Management Office, the People’s Government of Guangdong Province, Guangzhou, P. R. China; 11 Center of Inspection and Quarantine, School of Public Health, Sun Yat-sen University, Guangzhou, Guangdong Province, P. R. China; Santa Fe Institute, UNITED STATES

## Abstract

**Background:**

Dengue is a re-emerging infectious disease of humans, rapidly growing from endemic areas to dengue-free regions due to favorable conditions. In recent decades, Guangzhou has again suffered from several big outbreaks of dengue; as have its neighboring cities. This study aims to examine the impact of dengue epidemics in Guangzhou, China, and to develop a predictive model for Zhongshan based on local weather conditions and Guangzhou dengue surveillance information.

**Methods:**

We obtained weekly dengue case data from 1^st^ January, 2005 to 31^st^ December, 2014 for Guangzhou and Zhongshan city from the Chinese National Disease Surveillance Reporting System. Meteorological data was collected from the Zhongshan Weather Bureau and demographic data was collected from the Zhongshan Statistical Bureau. A negative binomial regression model with a log link function was used to analyze the relationship between weekly dengue cases in Guangzhou and Zhongshan, controlling for meteorological factors. Cross-correlation functions were applied to identify the time lags of the effect of each weather factor on weekly dengue cases. Models were validated using receiver operating characteristic (ROC) curves and k-fold cross-validation.

**Results:**

Our results showed that weekly dengue cases in Zhongshan were significantly associated with dengue cases in Guangzhou after the treatment of a 5 weeks prior moving average (Relative Risk (*RR*) = 2.016, 95% Confidence Interval (*CI*): 1.845–2.203), controlling for weather factors including minimum temperature, relative humidity, and rainfall. ROC curve analysis indicated our forecasting model performed well at different prediction thresholds, with 0.969 area under the receiver operating characteristic curve (AUC) for a threshold of 3 cases per week, 0.957 AUC for a threshold of 2 cases per week, and 0.938 AUC for a threshold of 1 case per week. Models established during k-fold cross-validation also had considerable AUC (average 0.938–0.967). The sensitivity and specificity obtained from k-fold cross-validation was 78.83% and 92.48% respectively, with a forecasting threshold of 3 cases per week; 91.17% and 91.39%, with a threshold of 2 cases; and 85.16% and 87.25% with a threshold of 1 case. The out-of-sample prediction for the epidemics in 2014 also showed satisfactory performance.

**Conclusion:**

Our study findings suggest that the occurrence of dengue outbreaks in Guangzhou could impact dengue outbreaks in Zhongshan under suitable weather conditions. Future studies should focus on developing integrated early warning systems for dengue transmission including local weather and human movement.

## Author Summary

Emerging and re-emerging infectious diseases in an urban city could expand due to increased urbanization, population density, and travel. Dengue, as a mosquito-borne viral disease, has rapidly spread from endemic areas to dengue-free regions, with social, demographic, entomological, and environmental factors affecting its transmission. In recent decades, Guangzhou has again suffered from several big outbreaks of dengue; as have its neighboring cities. In this study, we demonstrated that the dengue outbreaks in Guangzhou could impact outbreaks in Zhongshan, one of its neighboring cities, if suitable climate conditions are present. Such associations between dengue epidemics in two cities may also suggest the important role human movement has played in the transmission of the disease. Based on the association between dengue epidemics in Guangzhou and Zhongshan, and the association between dengue epidemics and weather conditions, we developed a reliable and robust model that predicts the occurrence of epidemics at diffrent thresholds in Zhongshan. These results could be used by local health departments in developing strategies towards dengue prevention and control, and push the public to pay more attention to social factors like human movement in disease transmission.

## Introduction

Currently, dengue is considered the most prevalent and rapidly growing mosquito-borne viral disease of humans [1], with 50% of the world’s population at risk [2]. In recent decades, it has spread from endemic areas, mainly tropical and subtropical regions, to dengue-free regions where social and environmental conditions were suitable [3]. Recent estimates of dengue include 390 million infections per year worldwide, including 96 million symptomatic [4]. There are currently no licensed vaccines or specific therapeutics, and the only way of controlling dengue is vector control, though with limited success [5]. However, interventions are still believed to be effective with increased resources [6]. In order to trigger timely interventions, alerts for potential outbreaks are of particular importance to mobilize vector control and to prime or reorganize healthcare services in preparation for a surge in dengue infections.

Determining the factors influencing the transmission of dengue is also important for helping to choose appropriate interventions. Dengue is usually transmitted by the bite of a mosquito infected with one of the four dengue virus serotypes. There are many factors involved in the transmission of dengue, including social, demographic, entomological, and environmental factors [1]. Temperature, humidity and rainfall all play important roles in mosquito biology and several studies have demonstrated a relationship between these variables and dengue transmission [7,8]. Additionally, due to the limited flight range of dengue mosquitoes, *Aedes aegypti* and *Aedes albopictus* [9,10], it has been recognized that human movement is another conclusive underlying driver of dengue virus dispersal at broad spatial scales such as between communities, regionally and globally [11–14], as well as at a narrow spatial scale from house to house [15,16].

Dengue re-emerged in mainland China in 1978, after more than 30 years’ absence. Since then, outbreaks and epidemics have been reported from time to time in varying scales, mainly in south China such as in Hainan province and Guangdong province [17]. In these periods, the two most severe outbreaks occurred in 1980 and 1986, before incidence rates of dengue became relatively stable and low after 1990 [17]. However, peak outbreaks were documented in Guangdong province again during 2013 (total 4,662 cases in mainland China, 62.08% in Guangdong province) and 2014 (total 48,162 cases, 93.83% in Guangdong province) [18–21]. Lai et al. [19] showed that the average annual incidence rate of dengue in mainland China from 1990 to 2014 was 2.2 cases per million people, while roughly calculated average annual incidence in Guangzhou reached as high as 29.14 cases per million from 2006 to 2013 [22–24], and 2888.57 cases per million in 2014 alone (37,305 indigenous cases [25] among 12,9368 million residents [26]). Average annual incidence rate of dengue in Zhongshan is also higher than the national level, with a rate of 30.46 cases per million people from 1990 to 2014, according to our estimate (S1 Table). In order to reduce dengue incidence in these cities, it is important to analyze the relationship of epidemics between these cities, to find any potential factor influencing the transmission of dengue and to set up an early warning system to initiate timely interventions.

In this study, we aimed to examine whether the dengue epidemics in Guangzhou can impact Zhongshan, a nearby city, and developed an early warning system for Zhongshan based on local weather conditions and dengue surveillance information in Guangzhou.

## Materials and Methods

### Study area

**Fig 1:**
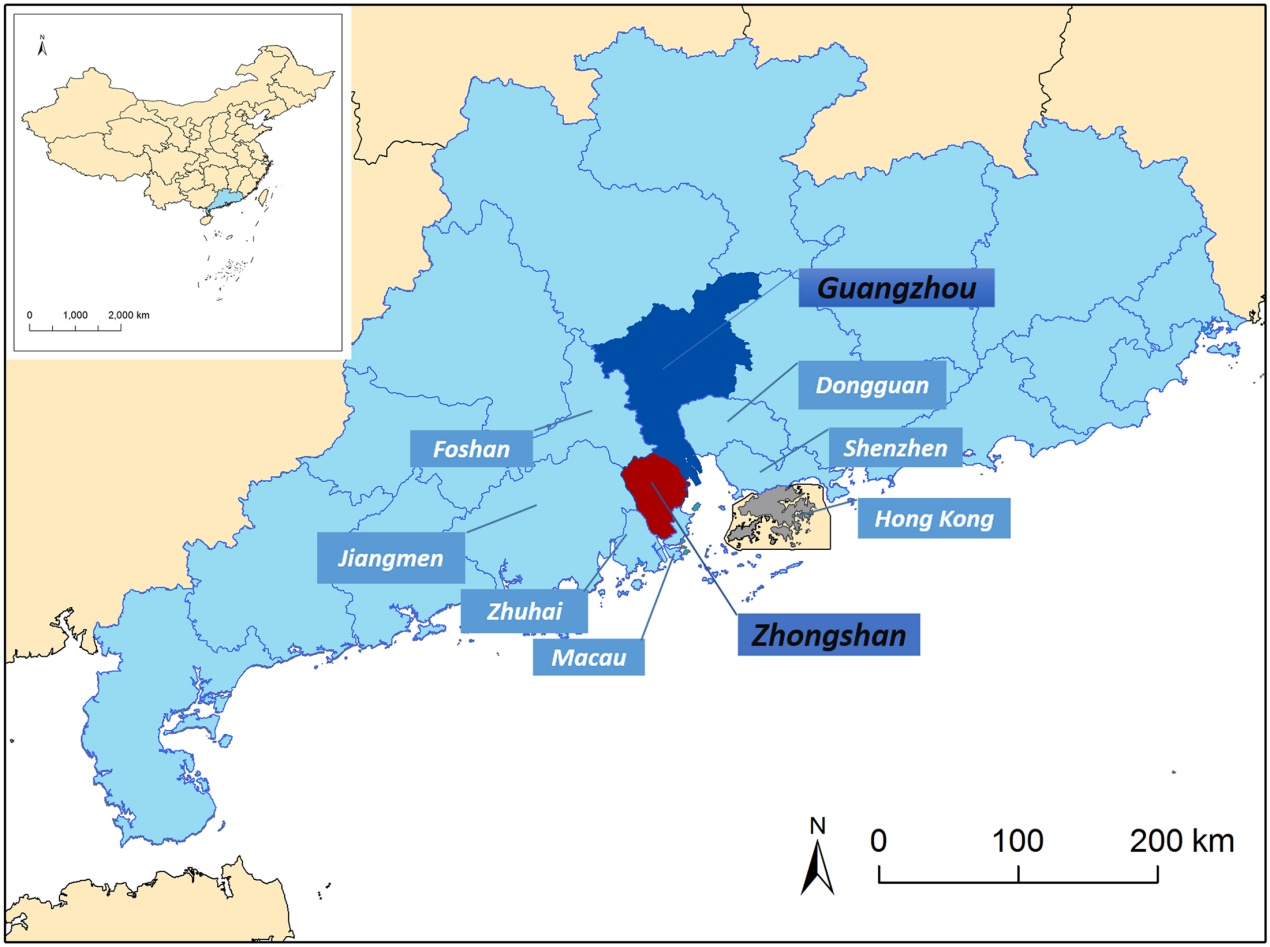
Locations of Guangzhou, Zhongshan and the neighboring cities. Nowadays, Guangzhou is considered a prominent commercial and business center in the PRD region. To maintain its role, major infrastructural projects were undertaken, including the construction of ring roads, highways, and railway tracks. This in turn provided easily accessible transportation between Guangzhou and other cities for the public. As Zhongshan is 86 kilometers from Guangzhou (a distance interconnected by Guangzhou-Zhuhai local train and several highways), workers and businessmen frequently travel between these two cities, and some go to work in Guangzhou during the day and return home to Zhongshan after work.

Guangzhou, located at the northern tip of the Pearl River Delta (PRD), is the provincial capital of Guangdong Province (Fig 1). Because of its location, Guangzhou possessed exceptional conditions as a port with a nickname “southern gate of China” [27]. Zhongshan, a medium-size city in Guangdong Province, adjacent to Guangzhou, is located along the west side of the mouth of the Pearl River, directly opposite Shenzhen and Hong Kong, south of Guangzhou and Foshan, east of Jiangmen, and north of Zhuhai and Macau (Fig 1), occupying an area of 1,800.14 square kilometers with 3.12 million permanent residents (Census reference 2010). It has a typical subtropical monsoon climate with hot and humid summer, mild to cool winter, monthly average temperature range from 13.8°C to 28.6°C, and annual rainfall of about 1,750 mm.

We selected these two cities as the study areas due to the high density of *Aedes albopictus*, the favorable climate, and the frequent outbreaks of dengue with recorded cases of both indigenous and imported cases [28–30].

### Data collection

Data on dengue cases in Guangzhou and Zhongshan for the period 1^st^ January 2005 to 31^st^ December 2014 were collected from the Chinese National Disease Surveillance Reporting System (NDSRS). The NDSRS is a web-based system set up in 2004 by the Chinese CDC. The system covers the entire population (1.3 billion people) living in all provinces, prefectures, and counties that make up mainland China. Thirty-nine reportable infectious diseases are currently included in this system. Individual cases, including clinically diagnosed and laboratory confirmed cases, are required to be reported by doctors within specified hours after diagnosis to the online system. Dengue has been included in the system as one of the surveilled diseases since 2005 [19], and dengue cases are required to be reported within 24 hours of diagnosis. Since dengue fever is a legally national notifiable infectious disease in China, case data was collected systematically and continuously. Dengue fever cases were diagnosed based on standardized laboratory tests and clinical/epidemiological investigations according to the Dengue Diagnostic Criteria enacted by the China Health Committee in 2001 and 2008. The data we collected from the NDSRS was anonymized. Overseas imported dengue cases were excluded in this study, as they did not represent the native transmission situation.

Meteorological data including weekly average minimum temperature, relative humidity, and rainfall for the period January 2005 to December 2014 in Zhongshan (S1 Fig) were collected from the Zhongshan Weather Bureau. Population data of Zhongshan (S2 Fig) was obtained from the Zhongshan Statistical Bureau.

### Data management and statistical analysis

In order to calculate the prevalence of dengue infections in the study areas, the time series of weekly case counts were plotted. In order to obtain a robust and smooth model, meteorological data was transformed into new variables using prior moving average of 5 weeks. Cross-correlations were also conducted between natural logarithm transformed weekly dengue case counts in Zhongshan (natural logarithm transformed case counts = *Ln* (case counts + 1)) and each new variable (the treated meteorological variable) to determine the best lag of meteorological parameter that leads to Zhongshan’s epidemics.

The epidemic data of Zhongshan (n = 522 weeks) was used as the dependent variable in the construction of log-linked negative binomial regression models. The model is one of the generalized linear models (GLMs) which can deal with count data to generate predition models in particular when data are over-dispersed (α>0), and where variance is larger than the mean [31]. The independent variables included were lagged weekly average minimum temperature, relative humidity, rainfall and an index based on the last 5 natural logarithm transformed weekly dengue case counts in Guangzhou in order to model weekly dengue case counts in Zhongshan. The natural logarithm of annual average population in Zhongshan was added as an offset variable. We ran several models with varying combinations of independent variables to obtain the final best-fit model. Here we only displayed the model with all four independent variables as follows:

Y~NB(r,p)

$$E\left(Y\right)=\mu =\frac{\text{r}(1-p)}{p}$$

$$Var(Y)=\mu +\frac{\mu^{2}}{\text{r}}$$

$$L\text{og}\left(\mu \right)=\beta_{0}+\text{log}\left(N\right)+\beta_{1}X_{1}+\beta_{2}X_{2}+\beta_{3}X_{3}+\beta_{4}X_{4}$$ (1)

Where *Y* is the observed weekly dengue case counts in Zhongshan; r and *p* are parameters of the negative binomial distribution; μ is the expectation of *Y*; β_0_ is the intercept; *N* is the annual population in Zhongshan so that log(*N*) is put as an offset in the model. *X*_*1*_, *X*_*2*_, *X*_*3*_ and *X*_*4*_ are the treated weekly dengue case counts in Guangzhou, weekly average minimum temperature, relative humidity and rainfall in Zhongshan, with β_1_, β_2_, β_3_, β_4_ as their coefficient vectors, respecitvely.

After we set up every possible model according to different combinations of the independent variables, we used the Akaike information criterion (AIC) [32], the first theoretic criterion to have gained widespread acceptance, to select the best-fit model, which can deal with the trade-off between the goodness of fit of the models and the complexity of the models.

Receiver operating characteristic (ROC) curve was used to validate our forecasting models at first. It can illustrate the performance of our models as a binary classifier at varied discrimination thresholds, and provide the most optimal cut-off for forecasting [33].

In order to validate the robustness of the model and avoid overfitting, k-fold cross-validation [34] was also applied. The original dataset was partitioned randomly into k subsets using random numbers generating from SPSS. For each cross-validation step, a single subset was retained as the test set, and the remaining k-1 subsets were used as the training set. The cross-validation process was then repeated k times with each of the k subsets used exactly once as the test set. The k results from the folds were then averaged to produce a single estimation. In this study, the k value is set to 10.

In addition, we used the samples from 1^th^ January 2005 to 27^th^ December 2013 (369 weeks in total) as training data and the samples from 28^th^ December 2013 to 26^th^ December 2014 (52 weeks in total) as test data to conduct another out-of-sample prediction.

The data management and statistical analyses were carried out using SPSS (version 19.0). All *p* values are two-sided and statistical significance was determined at the *p*<0.05 level.

## Results

During the study period, dengue epidemics varied annually in each of the study areas. Overall, dengue cases increased during the last decade, with maximum indigenous outbreaks in Zhongshan (n = 809) in 2013 and Guangzhou (n = 37,341) in 2014.

**Fig 2:**
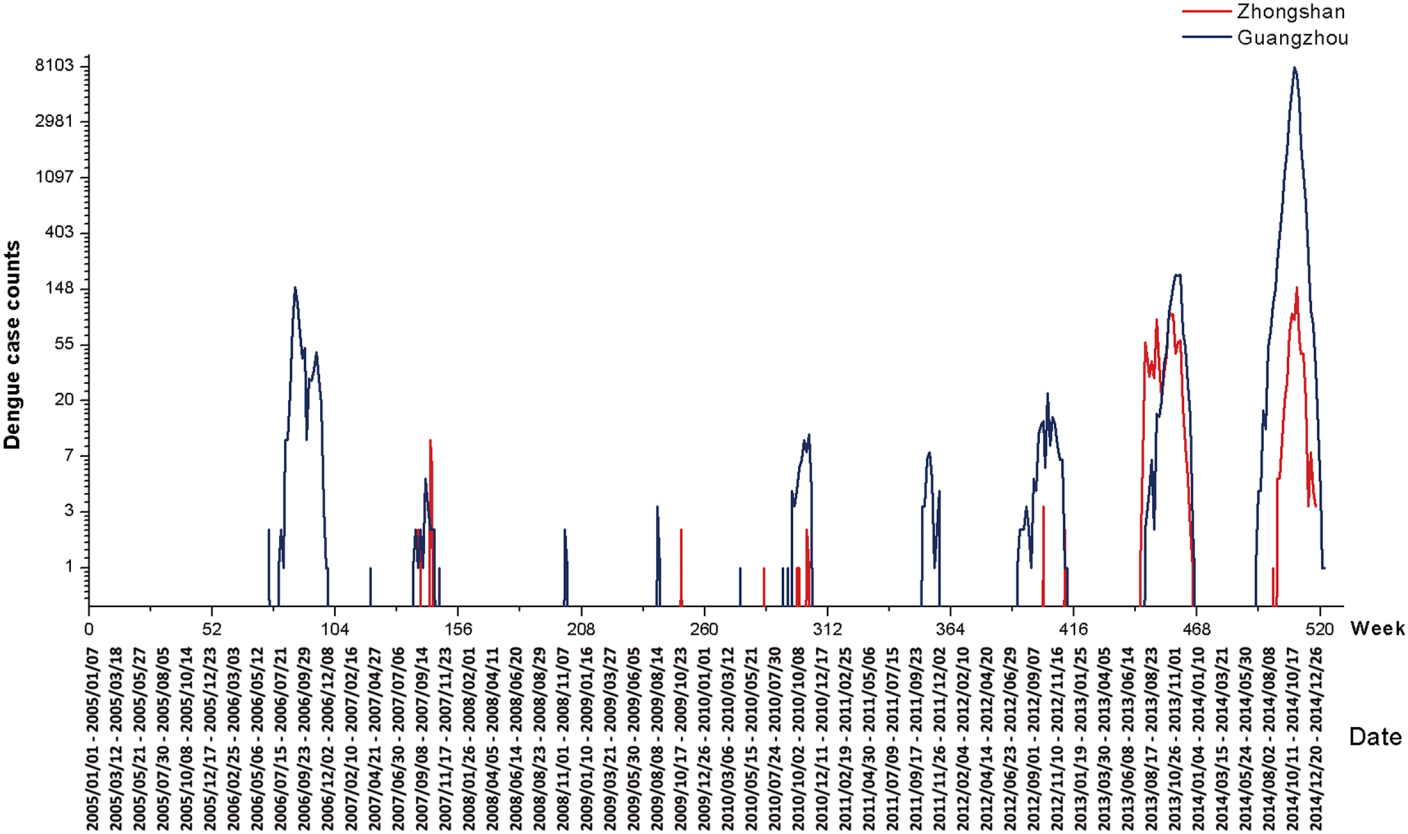
Weekly dengue case numbers in Zhongshan and Guangzhou, 2005 to 2014. * *: The Y axis is displayed in natural exponential scale.

There was at least one indigenous case in 25.48% (133 of 522 weeks) and 10.15% (53 of 522 weeks) of the observed weeks in Guangzhou and Zhongshan, respectively. The time series of weekly case counts (Fig 2) showed that dengue epidemics usually occurred in the summer and autumn months indicating that there was a seasonal pattern. Interestingly, regular epidemics in Zhongshan generally occurred after a few weeks of Guangzhou outbreaks, except for 2013.

### Cross-correlation

**Fig 3:**
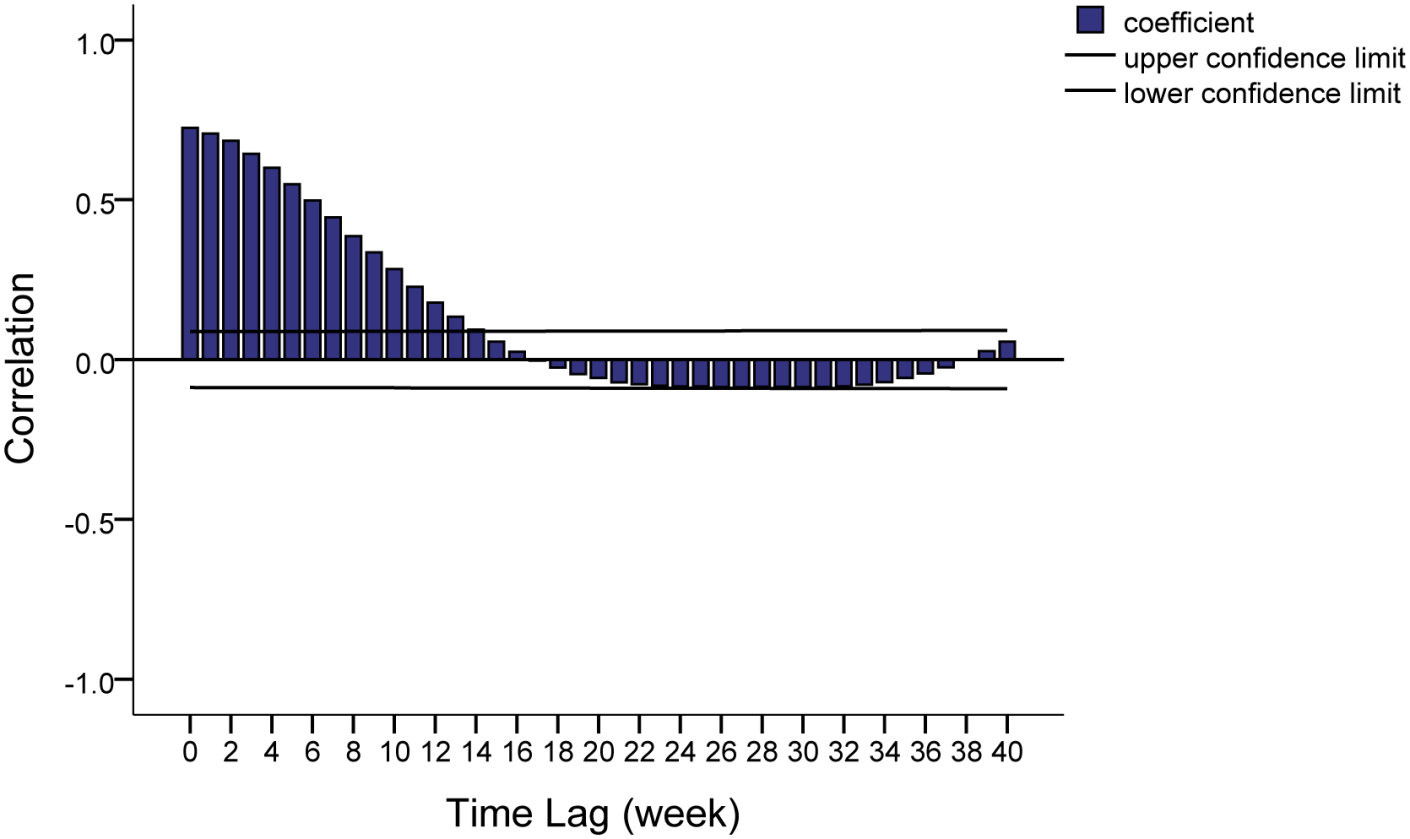
Cross correlations for natural logarithm transformed weekly dengue case counts of Zhongshan and Guangzhou. After making a prior moving average at a span of 5 weeks for each meteorological factor, cross-correlations were also used to find the best lags at which meteorological factors led to Zhongshan epidemics. Fig 4 shows that each treated variable was positively correlated with natural logarithm transformed weekly dengue cases in Zhongshan at different lags (Details in S2 Table). Therefore, we selected the nearest peak as the best lag for each variable. Treated weekly average minimum temperature, relative humidity and rainfall have triggered epidemics in Zhongshan by 6 weeks (correlation coefficient = 0.304), 15 weeks (correlation coefficient = 0.274) and 7 weeks (correlation coefficient = 0.299), respectively.

The result on the cross-correlations of natural logarithm transformed weekly case numbers of Zhongshan and Guangzhou (Fig 3) showed that the previous dengue epidemics in Guangzhou may have a great impact on the current week in Zhongshan, especially the last 5 weeks (correlation coefficient > 0.5). Taking this into consideration, an index was created based on the last 5 weekly Guangzhou case numbers via prior moving average, to be a predictor for forecasting epidemics in Zhongshan.

**Fig 4:**
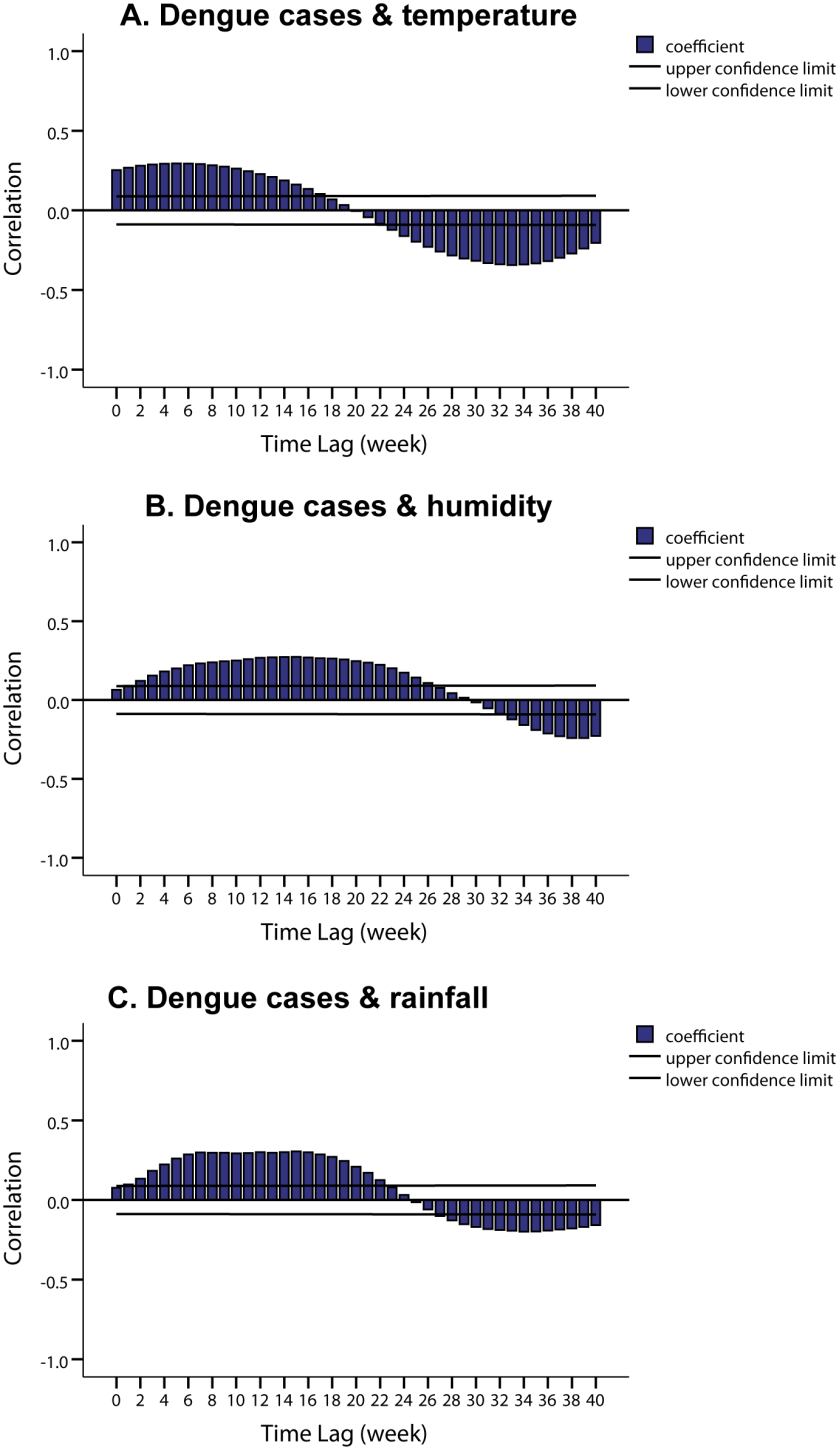
Cross-correlation plots for natural logarithm transformed weekly dengue case counts and different weekly meteorological factors in Zhongshan. A. Cross-correlation between natural logarithm transformed weekly dengue case counts and treated weekly average minimum temperature in Zhongshan, B. Cross-correlation between natural logarithm transformed weekly dengue case counts and treated weekly average relative humidity, C. Cross-correlation between natural logarithm transformed weekly dengue case counts and treated weekly accumulated rainfall.

### Model establishment

Table 1 showed the results on log-linked negative binomial regression models. The model including dengue cases in Guangzhou, minimum temperature, relative humidity, and rainfall was considered as the best-fit model (AIC = 841.442). In this model, the treated Guangzhou dengue cases had a possitive correlation with Zhangshan’s weekly dengue cases (Relative Risk (RR) = 2.016, 95% Confidence Interval (CI): 1.845–2.203). Similarly, treated minimum temperature (RR = 1.335, 95% *CI*: 1.125–1.583), treated relative humidity (1.207, 95% *CI*: 1.151–1.265), and treated rainfall (RR = 1.031, 95% *CI*: 1.026–1.037) also had possitive correlation with dengue case counts in Zhongshan.

**Table 1:** Regression models with different combination of the four candidate independent variables.

Model	Independent variables	AIC	RR	95% *CI*
1	dengue case counts in Guangzhou	1432.142	2.311	(2.120, 2.521)
2	minimum temperature	1240.207	3.118	(2.682, 3.626)
3	relative humidity	1811.849	1.256	(1.225, 1.288)
4	rainfall	1920.316	1.030	(1.026, 1.034)
5	dengue case counts in Guangzhou	1087.870	1.576	(1.451, 1.712)
	minimum temperature		2.062	(1.790, 2.375)
6	dengue case counts in Guangzhou	1129.243	1.838	(1.724, 1.959)
	relative humidity		1.299	(1.252, 1.347)
7	dengue case counts in Guangzhou	982.556	2.423	(2.202, 2.666)
	rainfall		1.035	(1.030, 1.039)
8	minimum temperature	1222.537	2.888	(2.487, 3.354)
	relative humidity		1.089	(1.049, 1.131)
9	minimum temperature	1179.341	3.505	(2.928, 4.196)
	rainfall		1.016	(1.011, 1.020)
10	relative humidity	1732.122	1.185	(1.154, 1.218)
	rainfall		1.016	(1.012, 1.020)
11	dengue case counts in Guangzhou	1033.461	1.582	(1.471, 1.700)
	minimum temperature		1.654	(1.445, 1.893)
	relative humidity		1.180	(1.129, 1.233)
12	dengue case counts in Guangzhou	903.789	1.942	(1.761, 2.142)
	minimum temperature		1.796	(1.506, 2.141)
	rainfall		1.028	(1.023, 1.033)
13	dengue case counts in Guangzhou	853.514	2.191	(2.025, 2.369)
	relative humidity		1.253	(1.200, 1.308)
	rainfall		1.034	(1.028, 1.039)
14	minimum temperature	1175.323	3.341	(2.788, 4.003)
	relative humidity		1.048	(1.009, 1.089)
	rainfall		1.014	(1.010, 1.019)
15	dengue case counts in Guangzhou	841.442	2.016	(1.845, 2.203)
	minimum temperature		1.335	(1.125, 2.203)
	relative humidity		1.207	(1.151, 1.265)
	rainfall		1.031	(1.026, 1.037)

### Model validation

**Fig 5:**
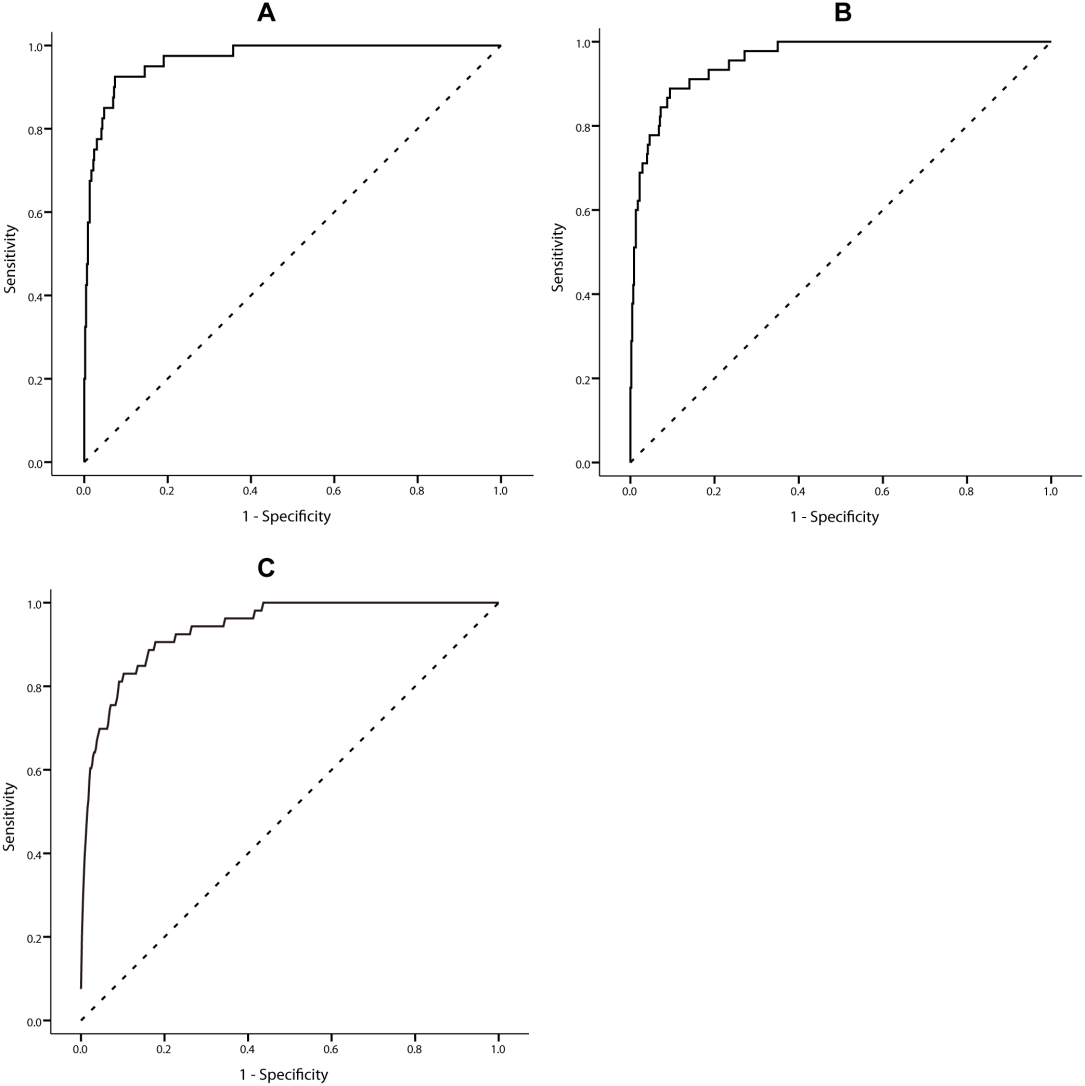
ROC curves for forecasting dengue epidemics in Zhongshan at a threshold of (A) 3 cases in a week, (B) 2 cases in a week and (C) 1 case in a week. To validate the robustness of our model and avoid overfitting, a k-fold cross-validation with a k value of 10 was applied. ROC curves were plotted again for each model in this section (S1 File), with average 0.938, 0.956 and 0.967 AUC at a forecasting threshold of 1, 2 and 3 cases per week, respectively. In the out-of-sample prediction (detailed results in S1 File), the sensitivity of our model remained as high as 78.83% and specificity 92.48% when we intend to forecast outbreaks involving more than 3 cases in a week in Zhongshan. Similarly, when we intend to forecast outbreaks with a smaller threshold, i.e., 1 case per week, our model had a sensitivity of 85.16% and specificity of 87.25% and at 2 cases per week, the sensitivity was 91.17% and specificity 84.00%. Such results meant that our model was rather robust and accurate.

When validated via ROC analysis using entire data, the forecasting model performed well in generating early warnings of dengue epidemics in Zhongshan at a reporting threshold of 3 cases in a single week, which means that the model can successfully estimate and announce epidemic risks of an outbreak involving ≥3 cases per week, with 0.969 area under the receiver operating characteristic curve (AUC), sensitivity of 92.5% and specificity of 92.6% (Fig 5A). Similarly, at a threshold of 2 cases per week, the forecasting model showed robust results, with 0.957 AUC, 88.9% sensitivity and 90.6% specificity; and at a threshold of 1 case per week, it had 0.938 AUC, 83.0% sensitivity and 90.0% specificity (Fig 5B and 5C).

**Fig 6:**
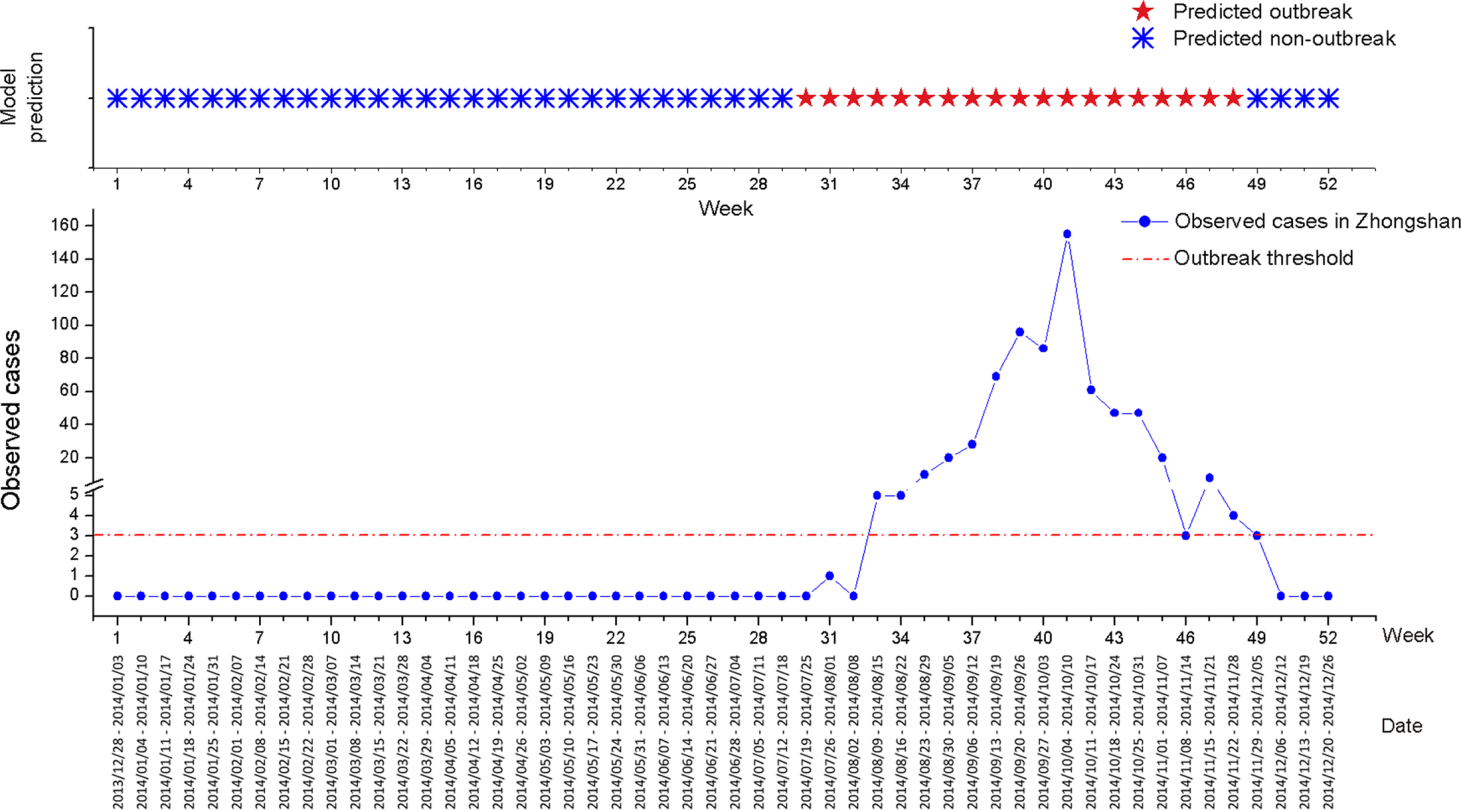
Out-of-sample prediction for dengue epidemics in Zhongshan (2013/12/28–2014/12/26).

The out-of-sample prediction for dengue epidemics in Zhongshan from 28^th^ December 2013 to 26^th^ December 2014 (52 weeks in total) was showed in Fig 6 and summarized in Table 2. The predictive model performed well in generating early warnings of dengue epidemics at the epidemic threshold of 3 cases per week, with 94.12% sensitivity and 91.43% specificity.

**Table 2:** Summary of out-of-sample prediction for dengue epidemics in Zhongshan (2013/12/28–2014/12/26).

Counts		Predicted outbreak		Total
		Yes	No	
Observed outbreak	Yes	16	1	17
	No	3	32	35
Total		19	33	52

## Discussion

This study assessed whether dengue epidemics in Guangzhou, China impact the epidemics in Zhongshan, and developed a predictive model for Zhongshan. Data on dengue cases, climate variables and demography for the years 2005 to 2014 were used in the modelling analysis.

Our research showed that weekly dengue cases in Zhongshan were significantly associated with dengue cases in Guangzhou after the treatment of 5 weeks prior moving average on natural logarithm transformation. Minimum temperature, relative humidity, and rainfall all had positive correlation with weekly dengue cases with different lags. This means that if the minimum temperature around 6 weeks before and the relative humidity around 15 weeks before was higher, and rainfall around 7 weeks before was heavier in Zhongshan, and if Guangzhou suffered from a bigger dengue epidemic in the last 5 weeks, the risk of a dengue epidemic in Zhongshan would be higher–and vice versa. ROC curve analysis and k-fold cross-validation suggested that our model performed well in generating early warnings of dengue epidemics in Zhongshan.

These findings indicated that the dengue epidemics in Guangzhou may be a potential predictor of dengue epidemics in Zhongshan, and demonstrated the effectiveness of evaluating the risk of suffering from emerging infectious diseases in one city on the basis of the situation in its neighboring cities, controlling other major risk factors such as local weather. The correlation between dengue epidemics in Guangzhou and Zhongshan also suggested the potential role that human movement has played in dengue transmission between cities, though this still needs further confirmation. Recently, Wesolowski, A. et al. have estimated flows of humans using cellular data in order to model and predict dengue epidemics in Pakistan [14]; Nunes, M. R. et al. have carried out a joint statistical analysis of evolutionary, epidemiological and ecological data and found that aerial transportation of humans and/or vector mosquitoes determine dengue virus spread in Brazil [12]. However, to our knowledge, our study is the first one to investigate the relationship between dengue epidemics in two cities directly and used it for epidemic forecasting in China.

The association of dengue epidemics between Guangzhou and Zhongshan is obvious–dengue epidemics in Guangzhou can predict following epidemics in Zhongshan when suitable climate is available, but the underlying reason may be more complicated than we thought. First, as indigenous dengue epidemics in South China are still regarded as a consequence of imported cases from overseas countries [35,36] and as Guangzhou is the “south gate of China” with significant international trade, Guangzhou may therefore have a higher risk of importing overseas cases at an earlier time than other cities, which makes Guangzhou serve as a sentinel for South China. For example, in 2014, the indigenous epidemic in Guangzhou was about 1.5 months earlier than that in Zhongshan. Second, Zhongshan has convenient transportation and close communication traditions with Guangzhou which allows easy transmission of cases between the two cities. As far as we know, lots of workers and businessmen are frequently traveling between these two cities, and some of them go to work in Guangzhou in day time but go back home in Zhongshan after work. In this way, dengue cases can easily be imported from Guangzhou to Zhongshan when the environment is appropriate, which suggested the role of human movement in dengue transmission. It has been showed that, among the first 40 imported dengue cases in Zhongshan in 2014, 31 of them were originally infected in Guangzhou. Certainly, the transmission of dengue may happen in both directions, but as we mentioned above, Guangzhou tends to suffer from dengue outbreaks more constantly and earlier, and there are more cases imported from Guangzhou to Zhongshan than that from Zhongshan to Guangzhou in the field. Zhongshan epidemic appeared to pre-date Guangzhou epidemic in 2013. One of the reasons might be that the weather and/or the mosquito density happened to be much more suitable for dengue transmission in Zhongshan in 2013 than that in Guangzhou. Therefore, further investigation is needed to identify the potential confounders in future research. Nevertheless, in general, epidemics were likely to occur earlier in Guangzhou, under most circumstances, than in Zhongshan.

Previous studies have demonstrated that climatic factors such as temperature, relative humidity and rainfall directly and/or indirectly influence dengue transmission [7,37]. Temperature impacts vector population development, reproductive rates [38], and extrinsic incubation period [39]. Additionally, temperature may also affect human behavior. For example, people will wear very light clothing when the temperature is high, which may increase the contact opportunities between the vector and human beings [35]. Rainfall provides mosquito breeding sites and stimulates egg hatching, thereby increasing mosquito population, while at the same time eliminating breeding sites through floods [40–42]. Humidity is another key factor that influences mosquitoes at different stages, especially during mating and egg laying. Studies have demonstrated that the combined effect of temperature and humidity significantly influences the number of blood meals and increases the survival rate of the vector [43]. Many studies have provided evidence to show the relationship between climatic factors and dengue transmission, but the intensity of the association varied with time and location in the Asia-Pacific region [44,45]. Temperature, relative humidity, and rainfall were major determinants of dengue transmission as meta analyses showed [44,45]. Many studies have also highlighted the importance of lag time of the climate variables [7,35,36,44,46–49]. For example, in Taiwan, there was a significant positive correlation with maximum temperature at lag 1–4 months, minimum temperature at lag 1–3 months, relative humidity at lag 1–3 months, and daily rainfall at lag 10 weeks [46]. In our study, minimum temperature, relative humidity, and rainfall led to an increase in dengue incidence in Zhongshan after about 6 weeks, 15 weeks, and 7 weeks, respectively, which is also consistent with the study in Taiwan [46]. The 6–7 weeks lag effect of minimum temperature and rainfall we observed is not unexpected. It accounts for the influence of climatic variables on the development, maturation, and survival of the *Aedes* mosquitoes (about 7–9 days from egg to adult) [50,51], as well as the extrinsic incubation period of DENV in the vector (10 days) [52] and the intrinsic incubation period in the human host (4–8 days) [1]. Moreover, the considerable life span of adult mosquitoes (around 3 weeks) provides multiple chances for the vectors to bite the humans [50,51]. However, relative humidity seems to have a positive impact on the epidemics at an earlier time in our research. The reason may lie in the fact that humidity influences mosquitoes especially at the early stage during mating and egg laying as mentioned above. The eggs can resist desiccation and withstand months of dormancy, and the duration and environmental humidity of the dormancy can affect larval survival, developmental rates and produces smaller adults [53].

We have applied treated data in our models as it is essential for a robust model. As dengue case counts is not normally distributed, we applied a natural logarithmic (ln) transformation for dengue cases to reduce the impact of dengue extreme values. For the modeling selection, due to the over-dispersion of the dengue cases relative to the Poisson distribution, we chose a generalized linear model with negative binomial-distributed and a log link. Morevoer, we tested and validated the model using different assumed distribution (eg., Poisson and negative binomial). Nagative binomial model gave a better fit in terms of a lower AIC value (AIC: range from 653.142 to 804.912), compared with the Possion model (AIC: range from 2348.906 to 2783.250). The use of prior moving average on weather variables can smooth out short-term fluctuations and highlight longer-term trends which also benefit our model because the impact of weather variables is usually mild and long-term. We performed cross-correlation analysis to assess the lagged correlations between climatic variables and dengue cases in Zhongshan, as literature suggests that the cross-correlation function can provide a statistical comparison of two sequences as a function of the time-shift between them [54]. Through this function, statistically significant correlations were found among several comparisons in our study, presenting relevant lags. And the results of the regression model confirmed these correlations furhter. We used ROC curve analysis to evaluate our predictive model as it took both sensitivity and specificity into account, and the area under the curve was considered as an effective measure of accuracy [55]. With the purpose to validate the robustness of the model and avoid overfitting, the k-fold cross-validation has properties of being simple and using all data for training and validation.

The limitations of this study should be acknowledged. First, reporting bias might be present as some of the health service centers or hospitals fail to report every case precisely due to political pressure or their lack of responsibility. However, as this study only included case data of 2005 to 2014, bias could be less or negligible. This is because the Chinese CDC has organised a surveillance diseases reporting system since 2005, with necessary measures implemented to improve reporting quality. Second, we have only included climatic variables but not mosquito density in this study. However, traditional indices of mosquito density such as House Index (HI), Container Index (CI), and Breteau Index (BI) require freguency surveillance, are not precise, and are highly dependent on both agent’s effort and householder availability [56], while new methods such as BG-Sentinel traps for adult mosquito surveillance have not been widely adopted in China. Some studies even found there was no or very weak relationship between traditional indices of *Aedes* mosquitoes and dengue incidence [57–59]. Finally, social-economic factors like environmental sanitation, average family income, education and human movement were not assessed in our study, as we believed that such factors would not have changed too much in Zhongshan during the study period, and data of human movement was not available temporarily.

### Conclusion

The study has demonstrated that dengue outbreaks in Guangzhou could impact outbreaks in Zhongshan, if suitable climate is available. A reliable and robust model, that predicts the occurrence of epidemics at a thredshold of 1, 2, or 3 dengue cases per week in Zhongshan was developed. The study results could be used by local health departments in developing strategies towards dengue prevention and control measures in Zhongshan. Future studies need to include social factors like human movement in the models.

## Supporting Information

S1 Fig Time series of meteorological factors in Zhongshan (2005–2014). (TIF)

S2 Fig Annual average population in Zhongshan (2005–2014). (TIF)

S1 Table Dengue incidence in Zhongshan (1990–2014). (DOCX)

S2 Table Cross-correlation coefficients for treated dengue cases in Zhongshan and four predicting factors. (DOCX)

S1 File ROC plots and out-of-sample prediction results during 10-fold cross-validation. (DOCX)
