# Supplementary figures and images for: Developing a Time Series Predictive Model for Dengue in Zhongshan, China Based on Weather and Guangzhou Dengue Surveillance Data
Source: PLoS Negl Trop Dis. 2016 Feb 19;10(2):e0004473. doi: 10.1371/journal.pntd.0004473 (PMC4764515; doi:10.1371/journal.pntd.0004473)

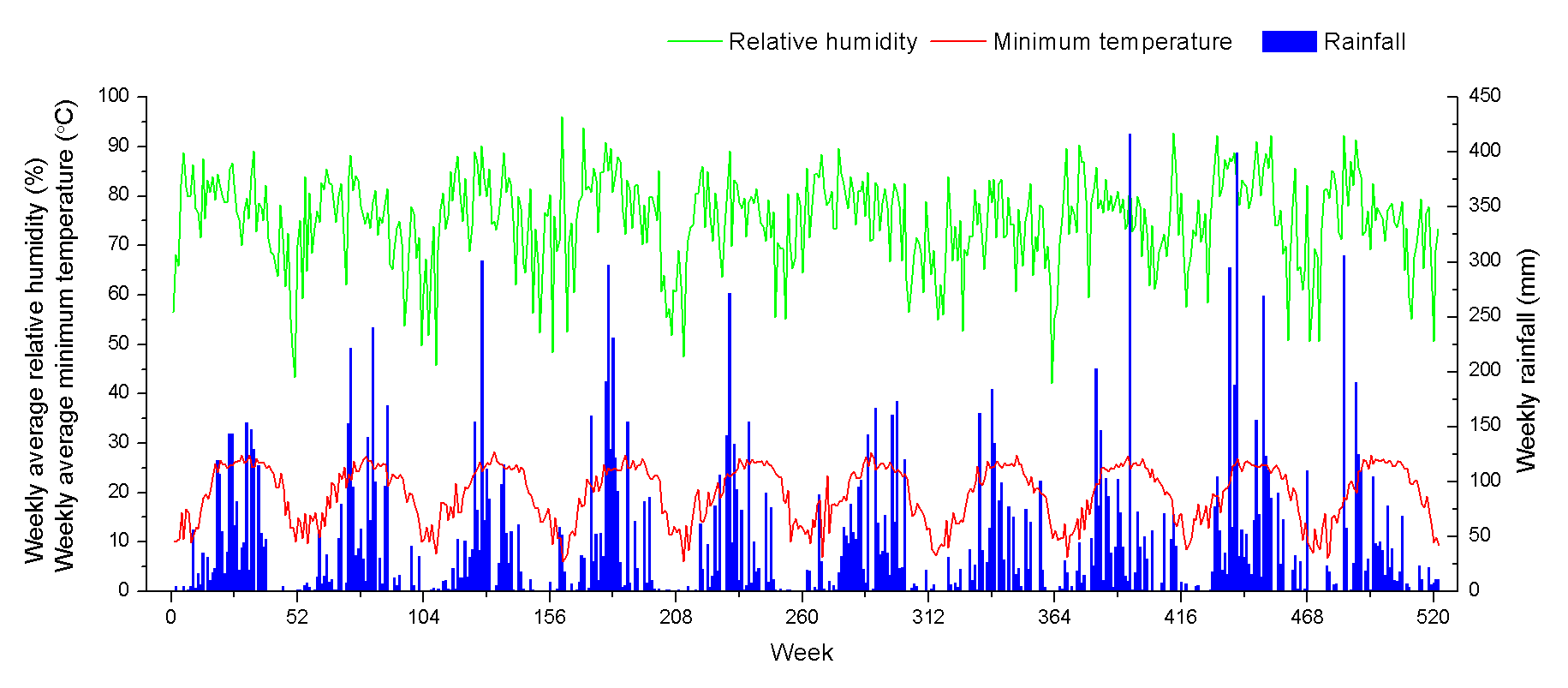

Supplement: S1 Fig — (TIF) [file pntd.0004473.s001.tif]

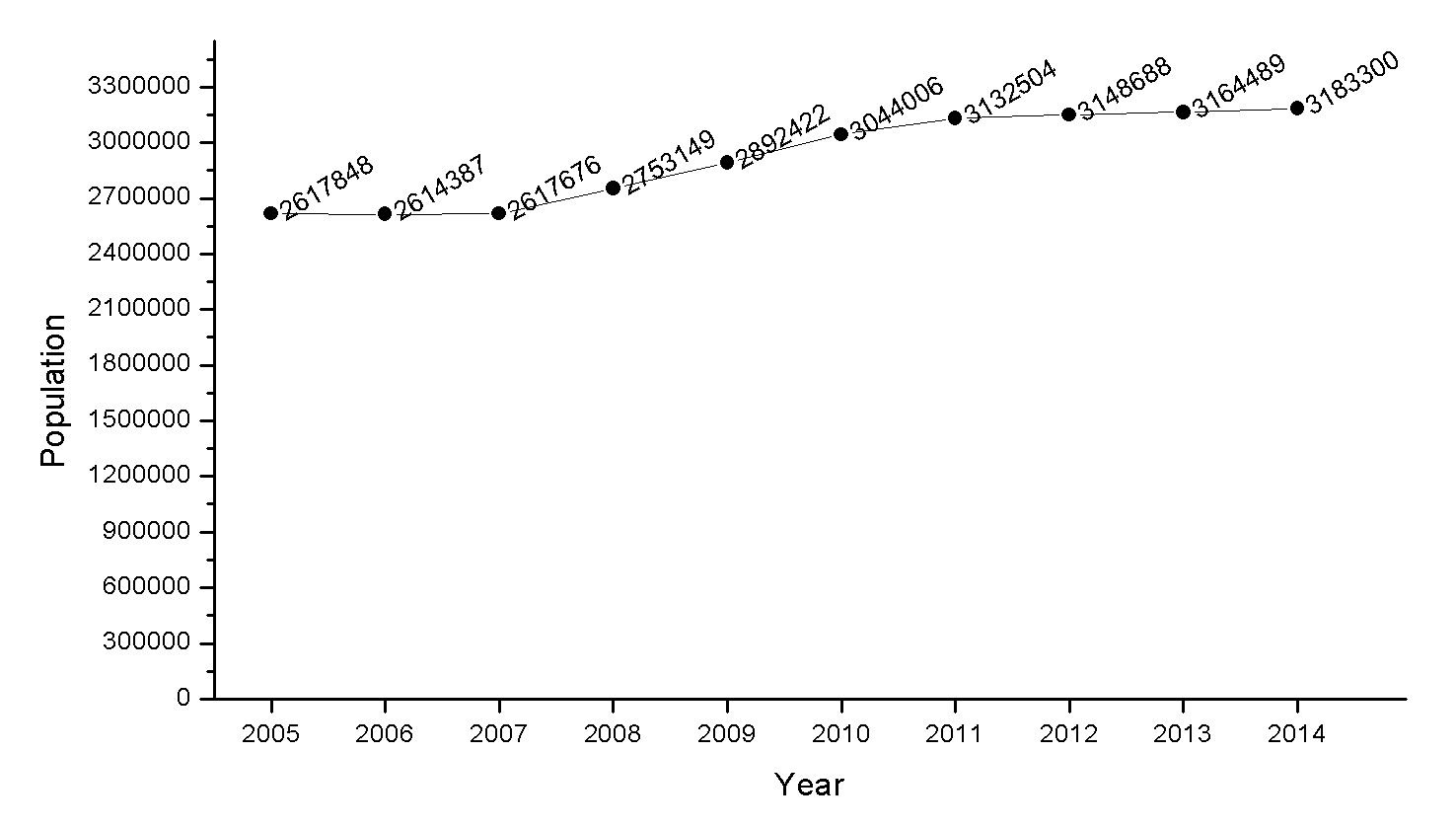

Supplement: S2 Fig — (TIF) [file pntd.0004473.s002.tif]
