# Supplementary material for: Developing a Time Series Predictive Model for Dengue in Zhongshan, China Based on Weather and Guangzhou Dengue Surveillance Data
Source: PLoS Negl Trop Dis. 2016 Feb 19;10(2):e0004473. doi: 10.1371/journal.pntd.0004473 (PMC4764515; doi:10.1371/journal.pntd.0004473)
Supplement: S1 Table — (DOCX) [file pntd.0004473.s003.docx]

| **S1 Table. Dengue incidence in Zhongshan (1990-2014)** | | | |
| --- | --- | --- | --- |
| Year | Dengue cases | Population | Incidence (per million) |
| 1990 | 0 | 1249200 | 0.00 |
| 1991 | 112 | 1290300 | 86.80 |
| 1992 | 0 | 1375250 | 0.00 |
| 1993 | 0 | 1465800 | 0.00 |
| 1994 | 0 | 1562300 | 0.00 |
| 1995 | 14 | 1665200 | 8.41 |
| 1996 | 0 | 1774850 | 0.00 |
| 1997 | 0 | 1774850 | 0.00 |
| 1998 | 0 | 2016250 | 0.00 |
| 1999 | 195 | 2149000 | 90.74 |
| 2000 | 0 | 2291100 | 0.00 |
| 2001 | 84 | 2373850 | 35.39 |
| 2002 | 28 | 2392150 | 11.70 |
| 2003 | 0 | 2410550 | 0.00 |
| 2004 | 38 | 2423550 | 15.68 |
| 2005 | 0 | 2687148 | 0.00 |
| 2006 | 0 | 2614387 | 0.00 |
| 2007 | 19 | 2617676 | 7.26 |
| 2008 | 1 | 2753149 | 0.36 |
| 2009 | 3 | 2892422 | 1.04 |
| 2010 | 4 | 3044006 | 1.31 |
| 2011 | 0 | 3132504 | 0.00 |
| 2012 | 5 | 3148688 | 1.59 |
| 2013 | 922 | 3164489 | 291.36 |
| 2014 | 668 | 3183300 | 209.85 |
| Average | / | / | 30.46 |
