# Supplementary material for: Developing a Time Series Predictive Model for Dengue in Zhongshan, China Based on Weather and Guangzhou Dengue Surveillance Data
Source: PLoS Negl Trop Dis. 2016 Feb 19;10(2):e0004473. doi: 10.1371/journal.pntd.0004473 (PMC4764515; doi:10.1371/journal.pntd.0004473)
Supplement: S2 Table — (DOCX) [file pntd.0004473.s004.docx]

| **S2 Table. Cross-correlation coefficients for treated dengue cases in Zhongshan and four predicting factors.** | | | | | | | | | | | |
| --- | --- | --- | --- | --- | --- | --- | --- | --- | --- | --- | --- |
| Lag (week) | Ln(ZS_cases) & Ln(GZ_cases) | |  | Ln(ZS_cases) & minimum temperature | |  | Ln(ZS_cases) & relative humidity | |  | Ln(ZS_cases) & rainfall | |
|  | Correlation coefficient | Std. error |  | Correlation coefficient | Std. error |  | Correlation coefficient | Std. error |  | Correlation coefficient | Std. error |
| 0 | 0.725 | 0.044 |  | 0.250 | 0.044 |  | 0.064 | 0.044 |  | 0.076 | 0.044 |
| 1 | 0.708 | 0.044 |  | 0.266 | 0.044 |  | 0.091 | 0.044 |  | 0.098 | 0.044 |
| 2 | 0.684 | 0.044 |  | 0.279 | 0.044 |  | 0.122 | 0.044 |  | 0.134 | 0.044 |
| 3 | 0.644 | 0.044 |  | 0.290 | 0.044 |  | 0.156 | 0.044 |  | 0.184 | 0.044 |
| 4 | 0.600 | 0.044 |  | 0.297 | 0.044 |  | 0.181 | 0.044 |  | 0.223 | 0.044 |
| 5 | 0.549 | 0.044 |  | 0.302 | 0.044 |  | 0.201 | 0.044 |  | 0.260 | 0.044 |
| 6 | 0.498 | 0.044 |  | 0.304 | 0.044 |  | 0.220 | 0.044 |  | 0.286 | 0.044 |
| 7 | 0.445 | 0.044 |  | 0.303 | 0.044 |  | 0.232 | 0.044 |  | 0.299 | 0.044 |
| 8 | 0.387 | 0.044 |  | 0.298 | 0.044 |  | 0.239 | 0.044 |  | 0.297 | 0.044 |
| 9 | 0.335 | 0.044 |  | 0.289 | 0.044 |  | 0.245 | 0.044 |  | 0.296 | 0.044 |
| 10 | 0.283 | 0.044 |  | 0.279 | 0.044 |  | 0.251 | 0.044 |  | 0.292 | 0.044 |
| 11 | 0.228 | 0.044 |  | 0.264 | 0.044 |  | 0.259 | 0.044 |  | 0.295 | 0.044 |
| 12 | 0.179 | 0.044 |  | 0.248 | 0.044 |  | 0.267 | 0.044 |  | 0.301 | 0.044 |
| 13 | 0.134 | 0.044 |  | 0.230 | 0.045 |  | 0.271 | 0.045 |  | 0.297 | 0.045 |
| 14 | 0.094 | 0.044 |  | 0.209 | 0.045 |  | 0.272 | 0.045 |  | 0.301 | 0.045 |
| 15 | 0.057 | 0.044 |  | 0.184 | 0.045 |  | 0.274 | 0.045 |  | 0.305 | 0.045 |
| 16 | 0.024 | 0.044 |  | 0.155 | 0.045 |  | 0.270 | 0.045 |  | 0.300 | 0.045 |
| 17 | -0.003 | 0.044 |  | 0.125 | 0.045 |  | 0.265 | 0.045 |  | 0.286 | 0.045 |
| 18 | -0.025 | 0.045 |  | 0.091 | 0.045 |  | 0.263 | 0.045 |  | 0.271 | 0.045 |
| 19 | -0.045 | 0.045 |  | 0.055 | 0.045 |  | 0.257 | 0.045 |  | 0.246 | 0.045 |
| 20 | -0.058 | 0.045 |  | 0.017 | 0.045 |  | 0.246 | 0.045 |  | 0.209 | 0.045 |
| 21 | -0.071 | 0.045 |  | -0.023 | 0.045 |  | 0.237 | 0.045 |  | 0.171 | 0.045 |
| 22 | -0.077 | 0.045 |  | -0.064 | 0.045 |  | 0.224 | 0.045 |  | 0.125 | 0.045 |
| 23 | -0.082 | 0.045 |  | -0.105 | 0.045 |  | 0.202 | 0.045 |  | 0.079 | 0.045 |
| 24 | -0.084 | 0.045 |  | -0.145 | 0.045 |  | 0.174 | 0.045 |  | 0.032 | 0.045 |
| 25 | -0.084 | 0.045 |  | -0.184 | 0.045 |  | 0.143 | 0.045 |  | -0.014 | 0.045 |
| 26 | -0.085 | 0.045 |  | -0.218 | 0.045 |  | 0.109 | 0.045 |  | -0.059 | 0.045 |
| 27 | -0.086 | 0.045 |  | -0.250 | 0.045 |  | 0.076 | 0.045 |  | -0.100 | 0.045 |
| 28 | -0.085 | 0.045 |  | -0.276 | 0.045 |  | 0.044 | 0.045 |  | -0.128 | 0.045 |
| 29 | -0.085 | 0.045 |  | -0.298 | 0.045 |  | 0.014 | 0.045 |  | -0.152 | 0.045 |
| 30 | -0.086 | 0.045 |  | -0.315 | 0.045 |  | -0.016 | 0.045 |  | -0.170 | 0.045 |
| 31 | -0.085 | 0.045 |  | -0.331 | 0.045 |  | -0.052 | 0.045 |  | -0.183 | 0.045 |
| 32 | -0.083 | 0.045 |  | -0.340 | 0.045 |  | -0.087 | 0.045 |  | -0.188 | 0.045 |
| 33 | -0.078 | 0.045 |  | -0.346 | 0.045 |  | -0.122 | 0.045 |  | -0.194 | 0.045 |
| 34 | -0.071 | 0.045 |  | -0.345 | 0.046 |  | -0.159 | 0.046 |  | -0.198 | 0.046 |
| 35 | -0.057 | 0.045 |  | -0.338 | 0.046 |  | -0.190 | 0.046 |  | -0.197 | 0.046 |
| 36 | -0.043 | 0.045 |  | -0.323 | 0.046 |  | -0.212 | 0.046 |  | -0.192 | 0.046 |
| 37 | -0.024 | 0.045 |  | -0.303 | 0.046 |  | -0.230 | 0.046 |  | -0.185 | 0.046 |
| 38 | 0.000 | 0.045 |  | -0.277 | 0.046 |  | -0.241 | 0.046 |  | -0.179 | 0.046 |
| 39 | 0.026 | 0.046 |  | -0.246 | 0.046 |  | -0.241 | 0.046 |  | -0.169 | 0.046 |
| 40 | 0.056 | 0.046 |  | -0.208 | 0.046 |  | -0.228 | 0.046 |  | -0.158 | 0.046 |
