# Supplementary material for: Developing a Time Series Predictive Model for Dengue in Zhongshan, China Based on Weather and Guangzhou Dengue Surveillance Data
Source: PLoS Negl Trop Dis. 2016 Feb 19;10(2):e0004473. doi: 10.1371/journal.pntd.0004473 (PMC4764515; doi:10.1371/journal.pntd.0004473)
Supplement: S1 File — (DOCX) [file pntd.0004473.s005.docx]

**S1 File. ROC plots and out-of-sample prediction results during 10-fold cross-validation.**

**Part 1.** ROC plots and out-of-sample prediction results for each model established during 10-fold cross-validation at the forecasting threshold of 1 case per week.

**Model 1 Model 2**


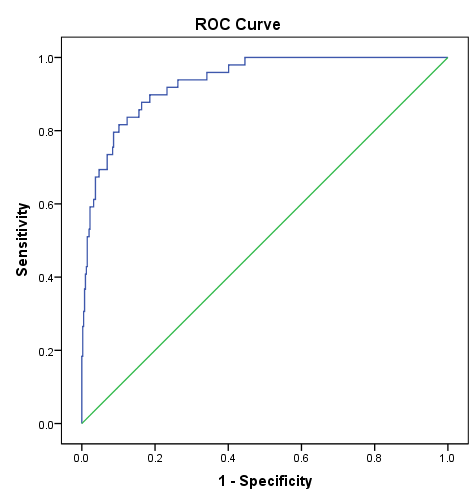

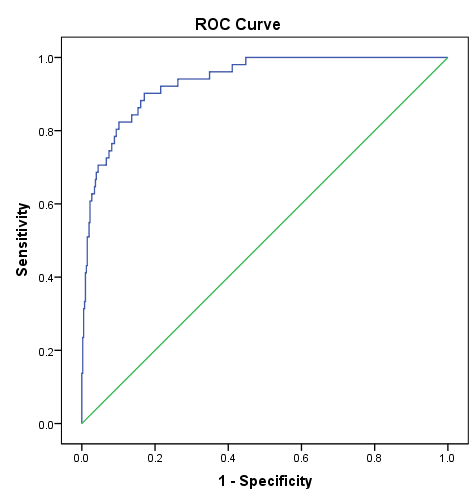


AUC=0.934 AUC=0.936

| **Table S1-1 Out-of-sample prediction** | | | | |  | **Table S1-2 Out-of-sample prediction** | | | | |
| --- | --- | --- | --- | --- | --- | --- | --- | --- | --- | --- |
| Count | | Prediction | | Total |  | Count | | Prediction | | Total |
|  |  | 0 | 1 |  |  |  |  | 0 | 1 |  |
| Outbreak | 0 | 43 | 5 | 48 |  | Outbreak | 0 | 39 | 11 | 50 |
|  | 1 | 0 | 4 | 4 |  |  | 1 | 0 | 2 | 2 |
| Total | | 43 | 9 | 52 |  | Total | | 39 | 13 | 52 |

Sensitivity (%) = 100.00 Sensitivity (%) = 100.00

Specificity (%) = 89.58 Specificity (%) = 78.00

**Model 3 Model 4**


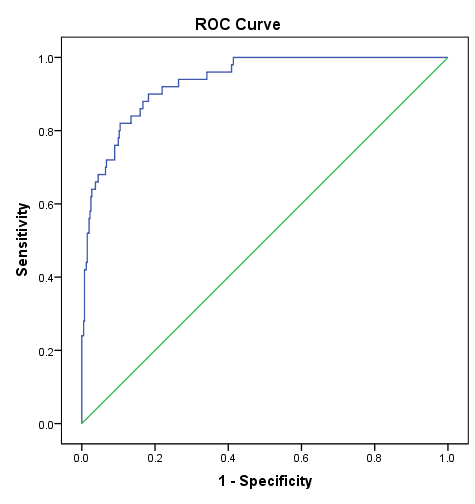

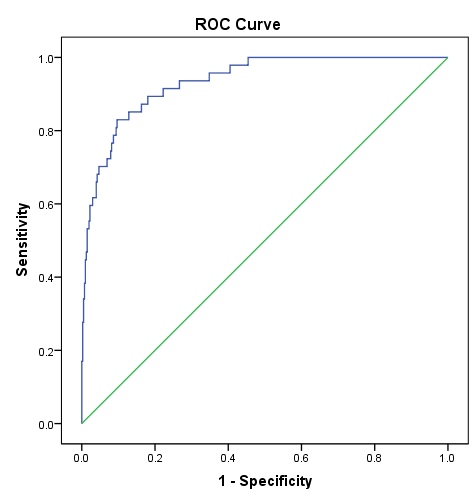


AUC=0.935 AUC=0.935

| **Table S1-3 Out-of-sample prediction** | | | | |  | **Table S1-4 Out-of-sample prediction** | | | | |
| --- | --- | --- | --- | --- | --- | --- | --- | --- | --- | --- |
| Count | | Prediction | | Total |  | Count | | Prediction | | Total |
|  |  | 0 | 1 |  |  |  |  | 0 | 1 |  |
| Outbreak | 0 | 41 | 8 | 49 |  | Outbreak | 0 | 43 | 3 | 46 |
|  | 1 | 0 | 3 | 3 |  |  | 1 | 1 | 5 | 6 |
| Total | | 41 | 11 | 52 |  | Total | | 44 | 8 | 52 |

Sensitivity (%) = 100.00 Sensitivity (%) = 83.33

Specificity (%) = 83.67 Specificity (%) = 93.47

**Model 5 Model 6**


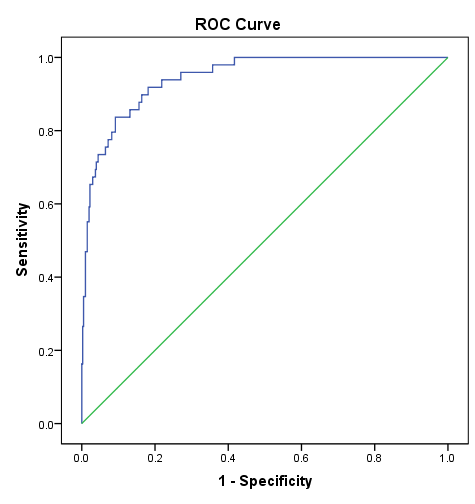

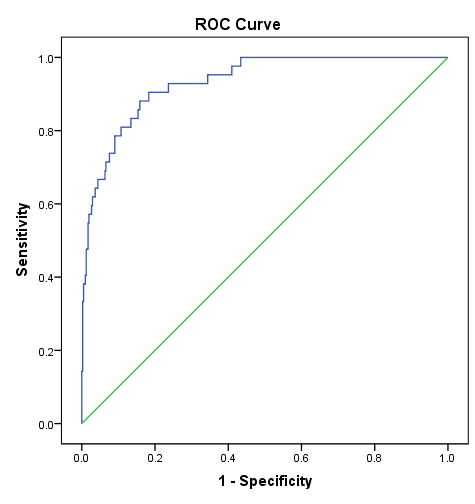


AUC=0.945 AUC=0.935

| **Table S1-5 Out-of-sample prediction** | | | | |  | **Table S1.6 Out-of-sample prediction** | | | | |
| --- | --- | --- | --- | --- | --- | --- | --- | --- | --- | --- |
| Count | | Prediction | | Total |  | Count | | Prediction | | Total |
|  |  | 0 | 1 |  |  |  |  | 0 | 1 |  |
| Outbreak | 0 | 45 | 3 | 48 |  | Outbreak | 0 | 35 | 6 | 41 |
|  | 1 | 2 | 2 | 4 |  |  | 1 | 1 | 10 | 11 |
| Total | | 47 | 5 | 52 |  | Total | | 36 | 16 | 52 |

Sensitivity (%) = 50.00 Sensitivity (%) = 90.91

Specificity (%) = 93.75 Specificity (%) = 85.37

**Model 7 Model 8**


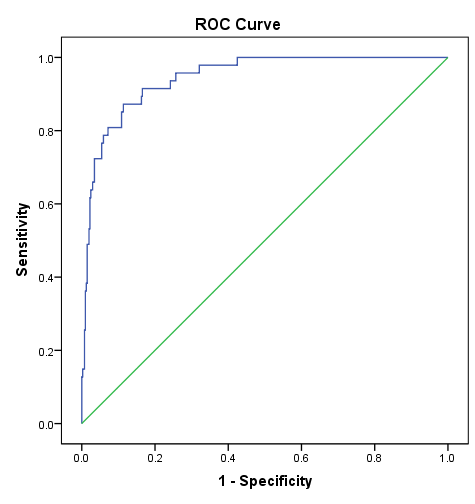

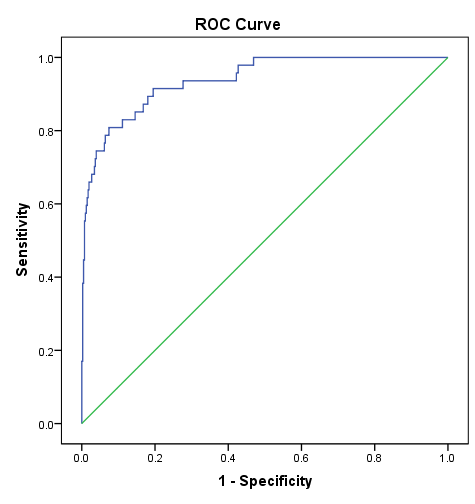


AUC=0.945 AUC=0.939

| **Table S1-7 Out-of-sample prediction** | | | | |  | **Table S1-8 Out-of-sample prediction** | | | | |
| --- | --- | --- | --- | --- | --- | --- | --- | --- | --- | --- |
| Count | | Prediction | | Total |  | Count | | Prediction | | Total |
|  |  | 0 | 1 |  |  |  |  | 0 | 1 |  |
| Outbreak | 0 | 40 | 4 | 44 |  | Outbreak | 0 | 43 | 3 | 46 |
|  | 1 | 0 | 8 | 8 |  |  | 1 | 2 | 4 | 6 |
| Total | | 40 | 12 | 52 |  | Total | | 45 | 7 | 52 |

Sensitivity (%) = 100.00 Sensitivity (%) = 66.67

Specificity (%) = 90.91 Specificity (%) = 93.47

**Model 9 Model 10**


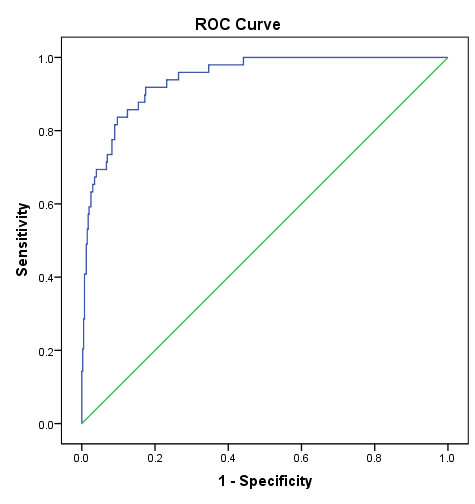

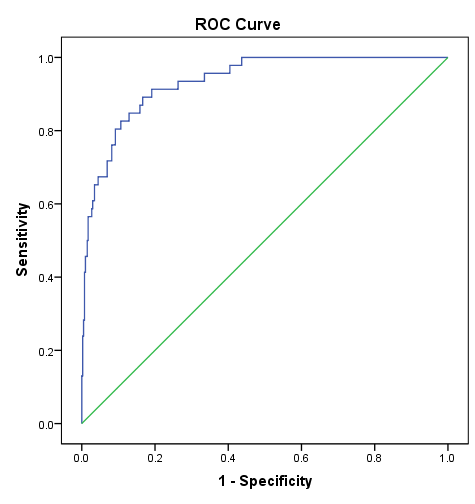


AUC=0.942 AUC=0.934

| **Table S1-9 Out-of-sample prediction** | | | | |  | **Table S1-10 Out-of-sample prediction** | | | | |
| --- | --- | --- | --- | --- | --- | --- | --- | --- | --- | --- |
| Count | | Prediction | | Total |  | Count | | Prediction | | Total |
|  |  | 0 | 1 |  |  |  |  | 0 | 1 |  |
| Outbreak | 0 | 38 | 10 | 48 |  | Outbreak | 0 | 40 | 7 | 47 |
|  | 1 | 1 | 3 | 4 |  |  | 1 | 1 | 6 | 7 |
| Total | | 39 | 13 | 52 |  | Total | | 41 | 13 | 52 |

Sensitivity (%) = 75.00 Sensitivity (%) = 85.71

Specificity (%) = 79.17 Specificity (%) = 85.11

Average AUC = 0.938

Average sensitivity (%) = 85.16

Average specificity (%) = 87.25

**Part 2.** ROC plots and out-of-sample prediction results for each model established during 10-fold cross-validation at the forecasting threshold of 2 cases per week.

**Model 1 Model 2**


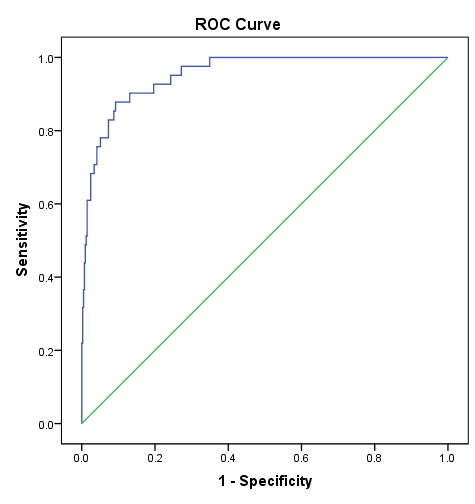

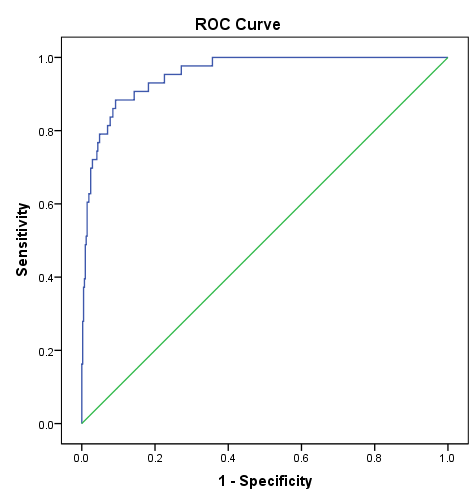


AUC=0.954 AUC=0.956

| **Table S2-1 Out-of-sample prediction** | | | | |  | **Table S2-2 Out-of-sample prediction** | | | | |
| --- | --- | --- | --- | --- | --- | --- | --- | --- | --- | --- |
| Count | | Prediction | | Total |  | Count | | Prediction | | Total |
|  |  | 0 | 1 |  |  |  |  | 0 | 1 |  |
| Outbreak | 0 | 43 | 5 | 48 |  | Outbreak | 0 | 48 | 2 | 50 |
|  | 1 | 0 | 4 | 4 |  |  | 1 | 1 | 1 | 2 |
| Total | | 43 | 9 | 52 |  | Total | | 49 | 3 | 52 |

Sensitivity (%) = 100.00 Sensitivity (%) = 50.00

Specificity (%) = 89.58 Specificity (%) = 96.00

**Model 3 Model 4**


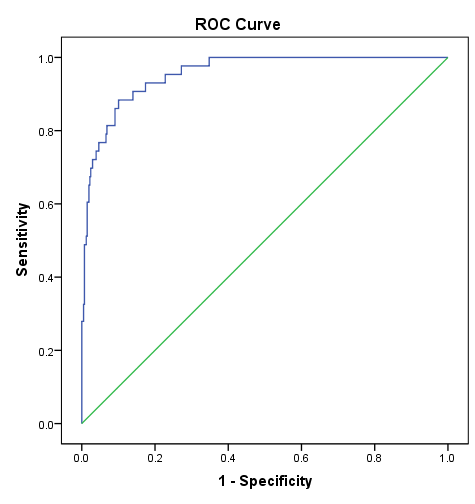

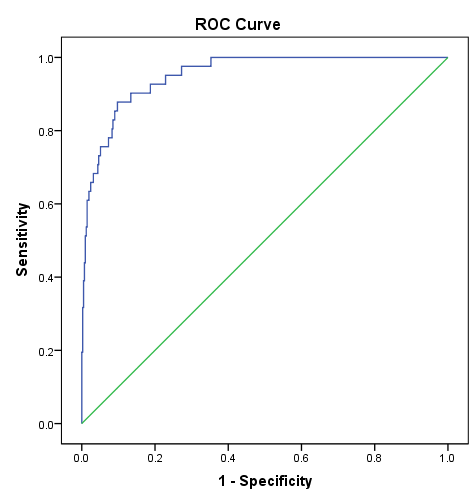


AUC=0.956 AUC=0.953

| **Table S2-3 Out-of-sample prediction** | | | | |  | **Table S2-4 Out-of-sample prediction** | | | | |
| --- | --- | --- | --- | --- | --- | --- | --- | --- | --- | --- |
| Count | | Prediction | | Total |  | Count | | Prediction | | Total |
|  |  | 0 | 1 |  |  |  |  | 0 | 1 |  |
| Outbreak | 0 | 46 | 4 | 50 |  | Outbreak | 0 | 45 | 3 | 48 |
|  | 1 | 0 | 2 | 2 |  |  | 1 | 0 | 4 | 4 |
| Total | | 46 | 6 | 52 |  | Total | | 45 | 7 | 52 |

Sensitivity (%) = 100.00 Sensitivity (%) = 100.00

Specificity (%) = 92.00 Specificity (%) = 93.75

**Model 5 Model 6**


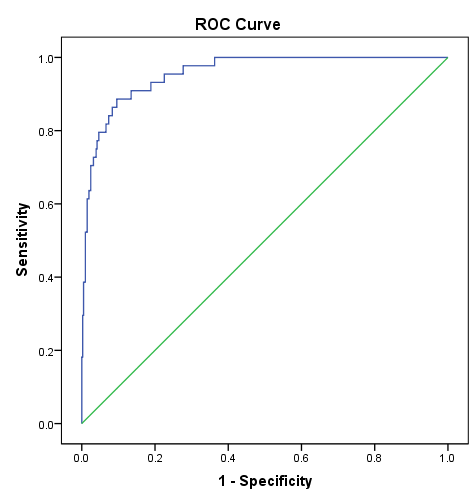

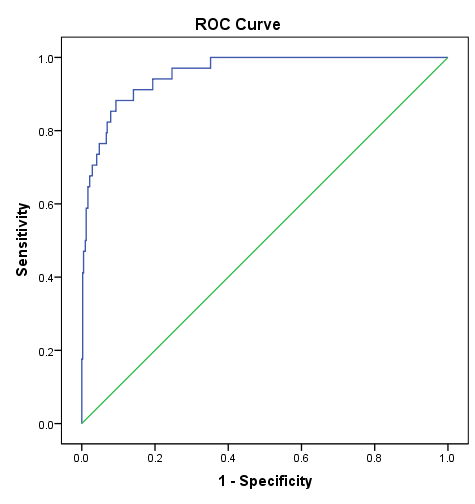


AUC=0.957 AUC=0.956

| **Table S2-5 Out-of-sample prediction** | | | | |  | **Table S2-6 Out-of-sample prediction** | | | | |
| --- | --- | --- | --- | --- | --- | --- | --- | --- | --- | --- |
| Count | | Prediction | | Total |  | Count | | Prediction | | Total |
|  |  | 0 | 1 |  |  |  |  | 0 | 1 |  |
| Outbreak | 0 | 47 | 4 | 51 |  | Outbreak | 0 | 38 | 3 | 41 |
|  | 1 | 0 | 1 | 1 |  |  | 1 | 1 | 10 | 11 |
| Total | | 47 | 5 | 52 |  | Total | | 39 | 13 | 52 |

Sensitivity (%) = 100.00 Sensitivity (%) = 90.91

Specificity (%) = 92.16 Specificity (%) = 92.68

**Model 7 Model 8**


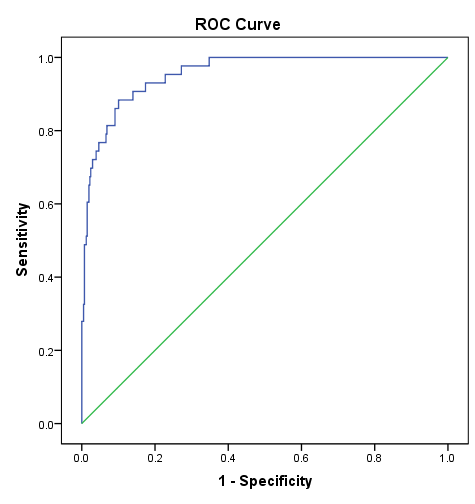

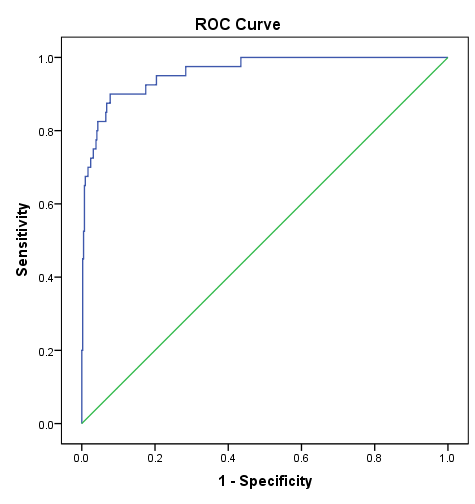


AUC=0.956 AUC=0.960

| **Table S2-7 Out-of-sample prediction** | | | | |  | **Table S2-8 Out-of-sample prediction** | | | | |
| --- | --- | --- | --- | --- | --- | --- | --- | --- | --- | --- |
| Count | | Prediction | | Total |  | Count | | Prediction | | Total |
|  |  | 0 | 1 |  |  |  |  | 0 | 1 |  |
| Outbreak | 0 | 38 | 8 | 46 |  | Outbreak | 0 | 44 | 3 | 47 |
|  | 1 | 4 | 2 | 6 |  |  | 1 | 1 | 4 | 5 |
| Total | | 42 | 10 | 52 |  | Total | | 45 | 7 | 52 |

Sensitivity (%) = 33.33 Sensitivity (%) = 80.00

Specificity (%) = 82.60 Specificity (%) = 93.62

**Model 9 Model 10**


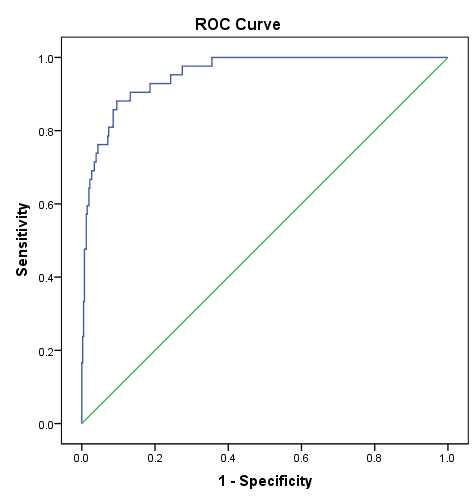

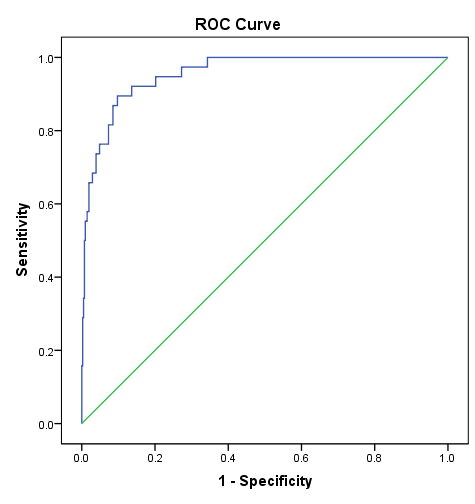


AUC=0.954 AUC=0.956

| **Table S2-9 Out-of-sample prediction** | | | | |  | **Table S2-10 Out-of-sample prediction** | | | | |
| --- | --- | --- | --- | --- | --- | --- | --- | --- | --- | --- |
| Count | | Prediction | | Total |  | Count | | Prediction | | Total |
|  |  | 0 | 1 |  |  |  |  | 0 | 1 |  |
| Outbreak | 0 | 42 | 7 | 49 |  | Outbreak | 0 | 44 | 3 | 47 |
|  | 1 | 0 | 3 | 3 |  |  | 1 | 1 | 6 | 7 |
| Total | | 42 | 10 | 52 |  | Total | | 45 | 9 | 54 |

Sensitivity (%) = 100.00 Sensitivity (%) = 85.71

Specificity (%) = 85.71 Specificity (%) = 93.62

Average AUC = 0.956

Average sensitivity (%) = 91.17

Average specificity (%) = 84.00

**Part 3.** ROC plots and out-of-sample prediction results for each model established during 10-fold cross-validation at the forecasting threshold of 3 cases per week.

**Model 1 Model 2**


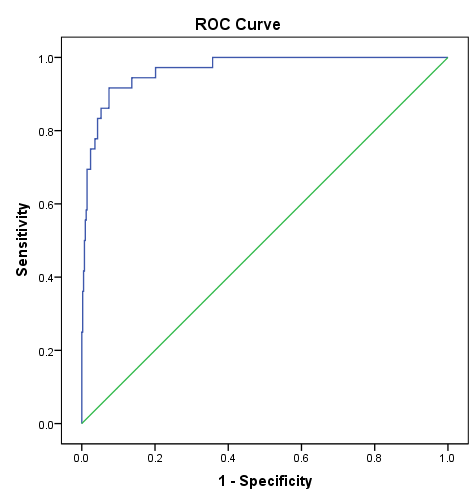

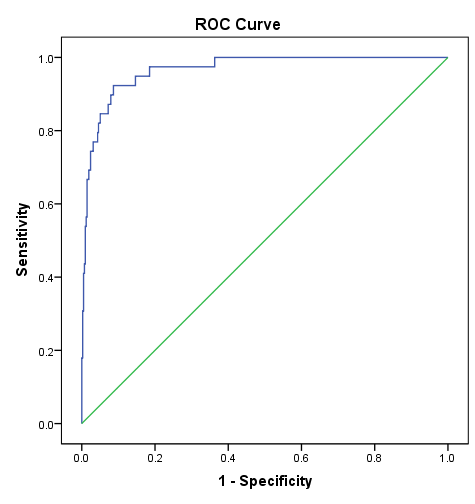


AUC=0.967 AUC=0.966

| **Table S3-1 Out-of-sample prediction** | | | | |  | **Table S3-2 Out-of-sample prediction** | | | | |
| --- | --- | --- | --- | --- | --- | --- | --- | --- | --- | --- |
| Count | | Prediction | | Total |  | Count | | Prediction | | Total |
|  |  | 0 | 1 |  |  |  |  | 0 | 1 |  |
| Outbreak | 0 | 46 | 2 | 48 |  | Outbreak | 0 | 49 | 2 | 51 |
|  | 1 | 1 | 3 | 4 |  |  | 1 | 0 | 1 | 1 |
| Total | | 47 | 5 | 52 |  | Total | | 49 | 3 | 52 |

Sensitivity (%) = 75.00 Sensitivity (%) = 100.00

Specificity (%) = 95.83 Specificity (%) = 96.08

**Model 3 Model 4**


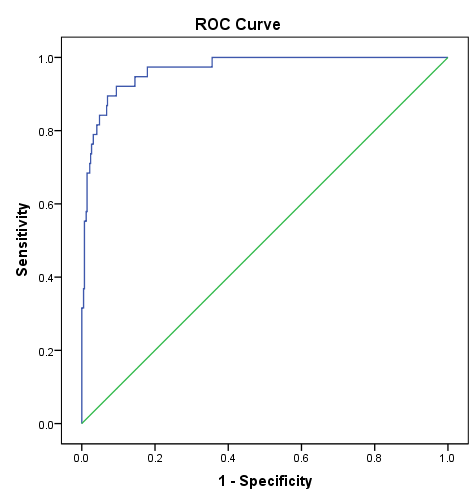

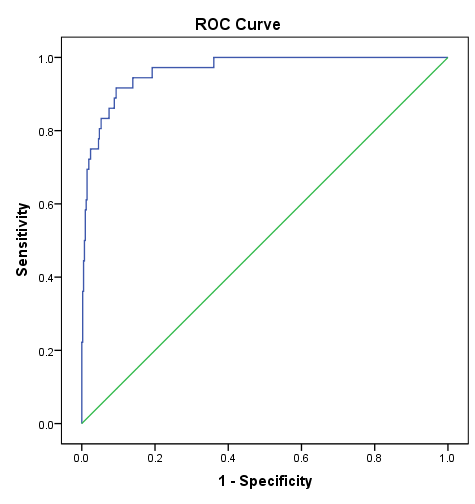


AUC=0.967 AUC=0.965

| **Table S3-2 Out-of-sample prediction** | | | | |  | **Table S3-3 Out-of-sample prediction** | | | | |
| --- | --- | --- | --- | --- | --- | --- | --- | --- | --- | --- |
| Count | | Prediction | | Total |  | Count | | Prediction | | Total |
|  |  | 0 | 1 |  |  |  |  | 0 | 1 |  |
| Outbreak | 0 | 46 | 4 | 50 |  | Outbreak | 0 | 46 | 2 | 48 |
|  | 1 | 0 | 2 | 2 |  |  | 1 | 0 | 4 | 4 |
| Total | | 46 | 6 | 52 |  | Total | | 46 | 6 | 52 |

Sensitivity (%) = 100.00 Sensitivity (%) = 100.00

Specificity (%) = 92.00 Specificity (%) = 95.83

**Model 5 Model 6**


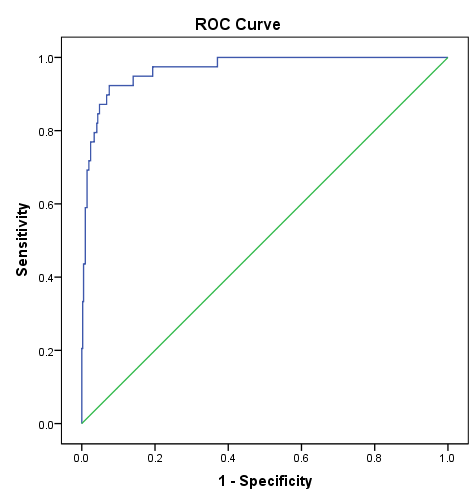

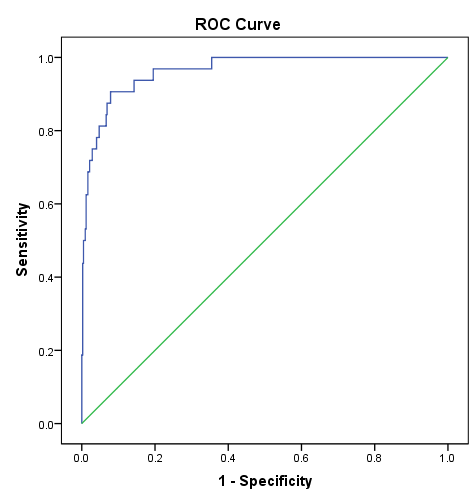


AUC=0.968 AUC=0.964

| **Table S3-5 Out-of-sample prediction** | | | | |  | **Table S3-6 Out-of-sample prediction** | | | | |
| --- | --- | --- | --- | --- | --- | --- | --- | --- | --- | --- |
| Count | | Prediction | | Total |  | Count | | Prediction | | Total |
|  |  | 0 | 1 |  |  |  |  | 0 | 1 |  |
| Outbreak | 0 | 47 | 4 | 51 |  | Outbreak | 0 | 40 | 4 | 44 |
|  | 1 | 1 | 0 | 1 |  |  | 1 | 0 | 8 | 8 |
| Total | | 48 | 4 | 52 |  | Total | | 40 | 12 | 52 |

Sensitivity (%) = 0.00 Sensitivity (%) = 100.00

Specificity (%) = 92.16 Specificity (%) = 90.91

**Model 7 Model 8**


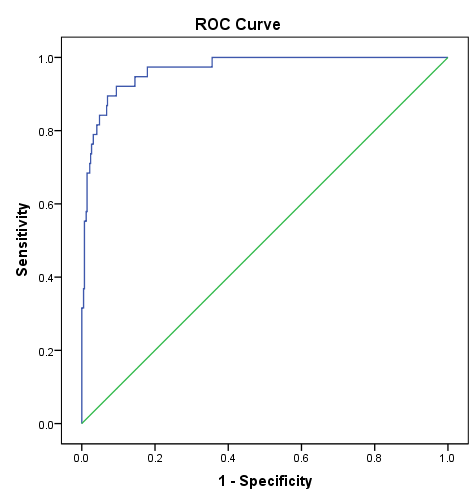

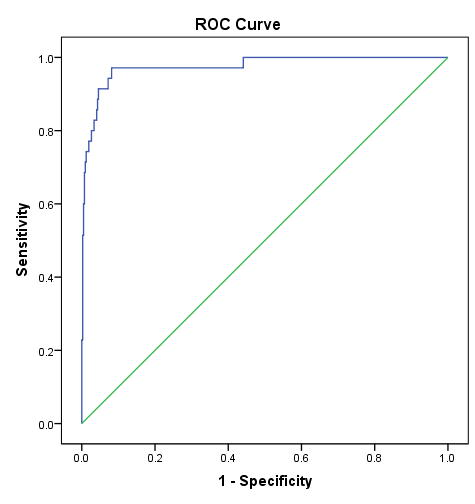


AUC=0.967 AUC=0.975

| **Table S3-7 Out-of-sample prediction** | | | | |  | **Table S3-8 Out-of-sample prediction** | | | | |
| --- | --- | --- | --- | --- | --- | --- | --- | --- | --- | --- |
| Count | | Prediction | | Total |  | Count | | Prediction | | Total |
|  |  | 0 | 1 |  |  |  |  | 0 | 1 |  |
| Outbreak | 0 | 39 | 7 | 46 |  | Outbreak | 0 | 44 | 3 | 47 |
|  | 1 | 4 | 2 | 6 |  |  | 1 | 1 | 4 | 5 |
| Total | | 43 | 9 | 52 |  | Total | | 45 | 7 | 52 |

Sensitivity (%) = 33.33 Sensitivity (%) = 80.00

Specificity (%) = 84.78 Specificity (%) = 93.62

**Model 9 Model 10**


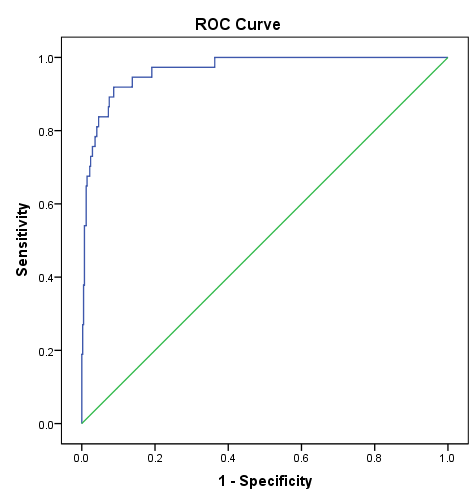

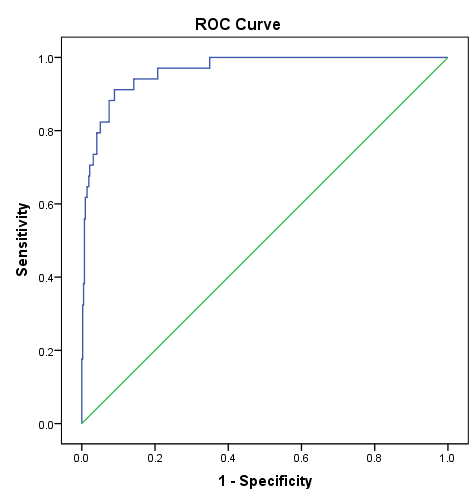


AUC=0.966 AUC=0.964

| **Table S3-9 Out-of-sample prediction** | | | | |  | **Table S3-10 Out-of-sample prediction** | | | | |
| --- | --- | --- | --- | --- | --- | --- | --- | --- | --- | --- |
| Count | | Prediction | | Total |  | Count | | Prediction | | Total |
|  |  | 0 | 1 |  |  |  |  | 0 | 1 |  |
| Outbreak | 0 | 44 | 5 | 49 |  | Outbreak | 0 | 45 | 3 | 48 |
|  | 1 | 0 | 3 | 3 |  |  | 1 | 0 | 6 | 6 |
| Total | | 44 | 8 | 52 |  | Total | | 45 | 9 | 54 |

Sensitivity (%) = 100.00 Sensitivity (%) = 100.00

Specificity (%) = 89.80 Specificity (%) = 93.75

Average AUC = 0.967

Average sensitivity (%) = 78.83

Average specificity (%) = 92.48
